# Microcapsule-Based Signal Amplification Method for Biomolecules

**DOI:** 10.3390/s19122711

**Published:** 2019-06-17

**Authors:** Masaki Yamaguchi

**Affiliations:** Graduate School of Medicine, Science & Technology, Shinshu University, 3-15-1 Tokida, Ueda, Nagano 386-8567, Japan; masakiy@shinshu-u.ac.jp; Tel.: +81-268-21-5444

**Keywords:** microcapsule, photo-responsive, signal simplification, micro-well array, biosensor

## Abstract

The direct signal amplification of target molecules could be an effective means of increasing the sensitivity and reducing the size of biosensors. The purpose of this study was to propose a novel signal amplification method suitable for the detection of biomolecules using microcapsules that can quickly respond to concentration variation. This microcapsule-based amplification method consists of two elements—microcapsules and a well-array. The microcapsules consist of (i) an inner shell fabricated through layer-by-layer assembly, (ii) a lipid bilayer, and (iii) loaded target molecules. In this method, the inner surface of the well-array was modified using TiO_2_ as a photocatalyst. The diameter and thickness of the fabricated micro-capsules for biomarker loading were shown to be 2.7 μm and 78 nm, respectively. An ultraviolet (UV) irradiation time of 5 min was needed when the change in optical density reached 90% saturation of the optical density change. Dye molecules were incorporated into the microcapsules and were subsequently released, and the concentration of the released solution changed in proportion with the encapsulated dye concentration. This demonstrates the proof of concept for this novel signal amplification method based on microcapsules.

## 1. Introduction

Various novel detection techniques for biomarkers have been proposed in order to enable improved sensitivity, sample preparation, chemical manipulation and reaction, high-throughput capabilities, and portability [1]. Most biosensors have three essential components, namely: a molecular recognition element interacting with the target analyte, a transducer element translating the biorecognition event into a useful electrical signal, and a microfluidic control mechanism consisting of several chambers connected by flow channels [2]. Quantitative bioassays show limited function with samples containing low concentrations of biomarkers. Consequently, signal amplification methods have been proposed, such as magnetic beads, microcantilevers, carbon nanotubes, or single-molecule digital counting assays [3,4,5,6]. The single-molecule digital counting assay is called a digital immunoassay technology or digital enzyme-linked immunosorbent assay (digital ELISA), and demonstrates detection sensitivity at a single-molecule level [7]. These digital counting devices include a micrometer-sized well array, with the concentration of the target molecule being estimated from the number of positive signal wells [8]. Digital immunoassay technology has improved the detection sensitivity to the 10^−16^ M range [9]. However, these amplification methods are not applicable to all types of biosensors, because they are not always compatible with the structure of the molecular recognition element or the transducer element of the sensor. Consequently, this may be considered one of the largest barriers to increasing the sensitivity and reducing the size of biosensors. If the direct signal amplification of target molecules is possible, it could contribute to the realization of point-of-care testing (POCT) systems for many clinically important biomarkers [10,11].

Microcapsules have attracted widespread interest because of their potential application in both chemical analysis and drug delivery [12,13]. A versatile microcapsule production method that allows for control over their size, stability, loading, and release properties is the layer-by-layer assembly technique [14,15,16,17]. Microcapsules incorporating a photocatalyst have been proposed for the UV-triggered release of encapsulated substances [18,19], and examples of this have been characterized as organic–inorganic hybrid vesicles, known as cerasomes [20]. Indeed, Angelatos et al. demonstrated that a target molecule can be captured on the antibody-modified surface of microcapsules, and the microcapsules can be optically triggered through irradiation with light to release the encapsulated substances [21]. Methods to modify the release rate of photo-responsive microcapsules were evaluated by Katagiri et al. through the variation of the SiO_2_/TiO_2_ composition of the outer shell; however, 1 h was needed in order to release the encapsulated molecules at a UV intensity of 5 mW/cm^2^ [22], which is too long to be of functional use within a bioassay. While these reaction properties might be characteristic of cerasomes, for the successful application of photo-responsive microcapsules in biosensors as a signal amplification method, a reduction in the reaction time to within several minutes would be required.

The aim of this study was to propose a proof-of-concept of a microcapsule-based amplification method that can respond quickly and is suitable for biomolecules, such as biomarkers. Using conventional photo-responsive microcapsules as a motif, a novel structure was designed, which consisted of microcapsules and a well-array with an inner-surface modified using TiO_2_ as a photocatalyst. The concept underlying this microcapsule-based amplification method was that the microcapsules will be quickly cleaved in the well-array through the scattering of the irradiated UV light in the wells.

## 2. Materials and Methods

### 2.1. Chemicals

Melamine-formaldehyde (MF) particle cores (custom-made; mean diameter: 2.7 ± 0.07 µm; concentration 10% *w*/*v*; microParticles GmbH, Berlin, Germany), polyethylenimine (PEI; 408700; CAS no. 9002-98-6; Merck KGaA, Darmstadt, Germany), poly(sodium 4-styrenesulfonate) (PSS; 527483; CAS no. 25704-18-1; Merck KGaA, Darmstadt, Germany), poly(diallyldimethylammonium chloride) (PDDA; 409014; CAS no. 26062-79-3; Merck KGaA, Darmstadt, Germany), and HCl (082-10095; CAS no. 7647-01-0; FUJIFILM Wako Pure Chemical Corporation, Osaka, Japan) were used to prepare the inner shell. Dioctadecyldimethylammonium bromide (DDAB; D1975; C_38_H_80_BrN; CAS no. 3700-67-2; Tokyo Chemical Industry Co., Tokyo, Japan) and toluene (34122-86; CAS no. 108-88-3; Nacalai Tesque Industry Co., Kyoto, Japan) were used to prepare the lipid bilayer. A TiO_2_-SiO_2_ coating (ST-K211; 0.5% TiO_2_ and 0.5% SiO_2_; Ishihara Sangyo Kaisha, Ltd., Osaka, Japan) was used as a photocatalyst. Phenol red solution (163-20623; 0.04% *w*/*v*; C_19_H_14_O_5_S; 36; 67; CAS no. 143-74-8; FUJIFILM Wako Pure Chemical Corporation, Osaka, Japan) was used for the evaluation of the microcapsule-based amplification method.

### 2.2. Principle and Structure

**Figure 1:**
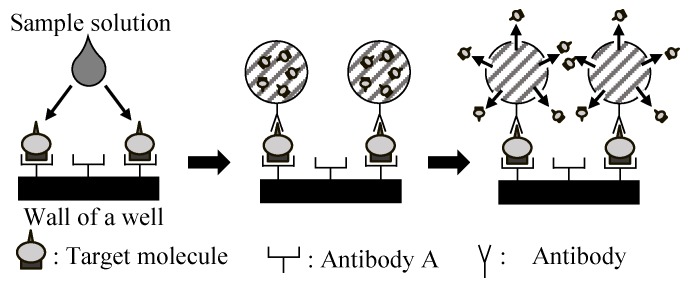
The conceptual principle underlying the direct signal amplification of the target molecules in the microcapsule-based amplification method.

Figure 1 shows the conceptual principle of the microcapsule-based amplification method. Firstly, the target molecules in a sample solution, such as blood, are trapped on the antibody-modified surface of the well-array. When the microcapsules are added to the well-array, each microcapsule is captured and sandwiches a target molecule. The microcapsule is then optically cleaved through irradiation with UV light so as to release the encapsulated substances. The amplitude can be controlled by the number of molecules contained within the microcapsules.

### 2.3. Fabrication of Photo-Responsive Microcapsules

**Figure 2:**
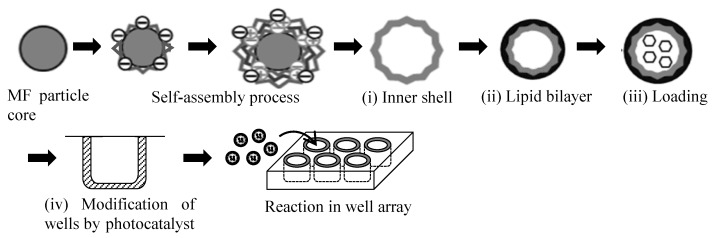
Schematic diagram of the preparation procedure for the two elements—the microcapsules and the well-array—needed for the microcapsule-based amplification method. MF—melamine-formaldehyde.

Figure 2 shows the preparation procedure for the two elements—the microcapsules and the well-array—needed for the microcapsule-based amplification method. The microcapsules consisted of (i) an inner shell, (ii) a lipid bilayer, and (iii) encapsulated molecules. The fabrication procedure for the microcapsules and the well-array were as follows.

(i) Inner shell: the inner shell was fabricated using alternating layers of polyanion (PSS) and polycation (PEI or PDDA) around a MF particle core [16,17]. Firstly, the MF particle cores were incubated in a PEI solution for 20 min (PEI was dissolved in 0.5 M NaCl at a 1 mg/mL concentration), followed by three cycles of centrifugation (2000 *g*; 3 min) with a supernatant exchange with distilled water followed by redispersion. PSS was adsorbed from a PSS solution for 5 min (PSS was dissolved in 0.5 M NaCl at 1 mg/mL) using the same procedure. The PDDA was subsequently adsorbed from a PDDA solution for 5 min (PDDA was dissolved in 0.5 M NaCl at 1 mg/mL) using the same procedure. The entire process was repeated five times so as to produce MF particles coated with five PSS/PDDA bilayers. Finally, this procedure was terminated with a PSS layer (MF/PEI(PSS/PDDA)_5_/PSS). The zeta (ζ) potential was measured so as to check the layer growth of the polyelectrolytes onto the MF particle core at each step using a zeta electrometer (ZEN3600, Zetasizer Nano ZS, Malvern Panalytical Ltd., Malvern, UK). A quartzcrystal microbalance (QCM, QCM2008-LVKIT, AFFINIX QNμ, ULVAC Inc., Kanagawa, Japan) was used to detect the mass changes from the frequency shift during the layer-by-layer assembly process. The resonance frequency, sample volume, and temperature during preparation were 27 MHz, 500 μL, and 24 °C, respectively. The thickness of the inner shell was estimated using a standard curve [23].

(ii) Lipid bilayer: DDAB powder was dissolved in toluene at 1 mM as a pretreatment. The solution was dried in a glass vial at 70 °C, and the top phase was separated carefully using a scraper. The scraped DDAB was re-dissolved in distilled water at 60 °C, which exceeded the phase transition temperature, and was dispersed using a supersonic wave homogenizer (LUH150, Yamato Scientific Co., Ltd., Tokyo, Japan) for 1 h. The MF particle core was removed through dissolution in 0.1 M of HCl. The microcapsules were subsequently coated with a cationic lipid bilayer of DDAB through three cycles of centrifugation (2000 *g*; 3 min) and a supernatant exchange with distilled water followed by redispersion. A lipid bilayer was produced in this manner (MF/PEI(PSS/PDDA)_5_/PSS/DDAB).

(iii) Loading of molecules into the microcapsules: phenol red, as a model target molecule, was post-loaded into the microcapsules by switching the lipid bilayer membrane permeability via temperature change. Microcapsules incubated in phenol red at 60 °C for 2 h (20 mL of microcapsule solution and 10 mL of phenol red solution) underwent three cycles of centrifugation (2000 *g*; 3 min) with supernatant exchange with distilled water, followed by redispersion. The images of each condition were observed using a scanning electron microscope (SEM; JSM-6010LA, Japan Electron Optics Laboratory Ltd., Tokyo, Japan) and a transmission electron microscope (TEM; JEM-2100, Japan Electron Optics Laboratory Ltd., Tokyo, Japan).

(iv) Modification of photocatalyst in the wells: a photocatalyst layer was immobilized on the wall of the well-array (3801-096, Iwaki Co., Tokyo, Japan) using a TiO_2_-SiO_2_ coating. The volume and surface area of each well were 403.8 μL and 271.5 mm^2^, respectively. An aliquot (360 μL) of the TiO_2_-SiO_2_ coating was added to each well to fill 90% of the well volume, and they were dried at 24 °C for 2 h. A UV light (MSPT-UV3; 365 nm wavelength; MeCan Imaging Inc., Fujimino, Japan) was used to cleave the microcapsules optically.

### 2.4. Evaluation of the Photo-Responsive Microcapsules-Based Amplification Method

#### 2.4.1. Time-Course Changes

The time-course changes in the concentration of the encapsulated molecules released from the microcapsules were evaluated. Phenol red (1.13 mM) was used as the target molecule. The time-course changes in optical density (OD) were measured using a spectrophotometer (U-3010, Hitachi Ltd., Tokyo, Japan). Phenol red was loaded into the microcapsules and cleaved through irradiation with 510 mW/cm^2^ (mean value) of UV light (MSPT-UV3, 365 nm wavelength, MeCan Imaging Inc., Fujimino, Japan). The concentration of the loaded phenol red was estimated from the loading procedure to be 5.65 μM. As a control, a phenol red solution was assessed at the same concentration. The optical density at each time point was shown as the change rate (ΔOD), representing the value of difference from 0 min.

#### 2.4.2. Concentration Characteristics

The concentration of the encapsulated and released phenol red was evaluated. Four different concentrations of phenol red (ρ), 0.13, 0.25, 0.31, and 0.38 mM, were used to load the microcapsules by mixing the microcapsule solution at different ratios. The optical densities were measured using the same procedure. The concentration ratios (*x*) were calculated based on the OD at 0.13 mM.

## 3. Results and Discussion

### 3.1. Fabrication of Photo-Responsive Microcapsules

**Figure 3:**
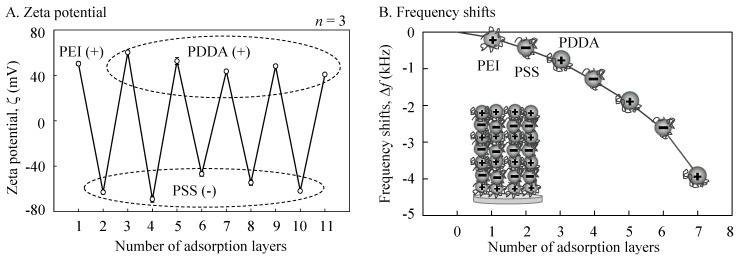
Measured results of frequency shifts for the alternate adsorption of poly(sodium 4-styrenesulfonate) (PSS)/poly(diallyldimethylammonium chloride) (PDDA) on the MF particle cores at 0.5 M of each solvent for 5 min. (**A**) Zeta potential; (**B**) Frequency shifts.

The results of the zeta potential were measured for each of the five steps of the PSS/PDDA layering procedure (Figure 3A). For the polyelectrolytes, electrophoresis yielded a complete charge reversal at each step. The QCM frequency decreased proportionally upon an increase in mass (Figure 3B). The thickness of the inner shell (d) was estimated to be 78 nm in total (d = 0.02 (−ΔF) nm). It was considered that the alternate layering using oppositely charged polycations was successfully performed so as to produce anionic cerasomes.

**Figure 4:**
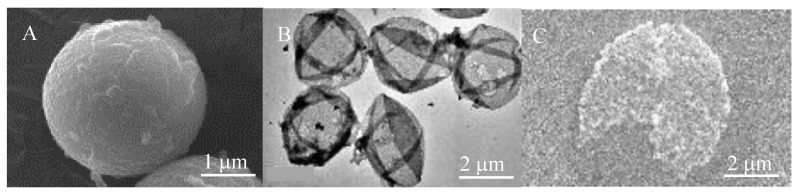
Fabricated microcapsules. (**A**) SEM image of the MF particle core. (**B**) TEM image of the inner shell after the removal of the MF particle core. (**C**) SEM image of particle after UV irradiation.

Figure 4 shows the SEM (A and C) and TEM (B) analyses of the microcapsules performed before and after UV exposure of the well-array (MF particle core (A); the inner shell after removal of the MF particle core (PSS/PDDA)_5_/PSS) (B); and the particle after UV irradiation (C)). Prior to UV irradiation, the particles had a spherical structure for both the inner shell and the lipid bilayer capsules, with the same diameter as the MF particle core (2.7 µm). It was considered that the templating MF particle cores (Figure 4A) had decomposed following HCl treatment, to yield PSS/PDDA multilayer microcapsules (Figure 4B), because the microcapsules were completely crushed by UV irradiation (Figure 4C; 510 mW/cm^2^).

### 3.2. Evaluation of Photo-Responsive Microcapsules-Based Amplification Method

**Figure 5:**
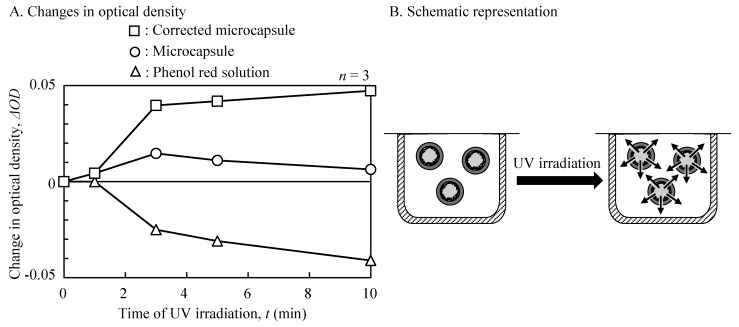
Time-course changes of optical densities for UV irradiation (wavelength 430 nm). ΔOD—change rate of the optical density. (**A**) Changes in optical density; (**B**) Schematic representation.

The time-course changes in the release of the encapsulated molecules from the fabricated microcapsules were evaluated using the low molecular weight dye (phenol red) and a photocatalyst-modified well-array (two wells). The mean UV intensity was fixed at 510 mW/cm^2^ (800 mW/cm^2^ maximum). Figure 5 shows the changes in the optical densities (430 nm) with the UV irradiation time. Before UV irradiation, the optical density of the microcapsules showed a minimum value (0.474), reflecting virtually no dye release. Following irradiation, the optical density of the microcapsules increased over time, before decreasing to the initial levels. To investigate this phenomenon, the optical density of a phenol red solution alone was measured, following UV irradiation as a control. Prior to UV irradiation, the optical density of the phenol red solution showed 0.624. Following irradiation, a negative increase in the change rate (ΔOD) of phenol red was observed. Following a correction for phenol red fading, the time-course changes of the optical densities in the microcapsule were calculated. The corrected optical density was saturated to 90% at 5 min.

**Figure 6:**
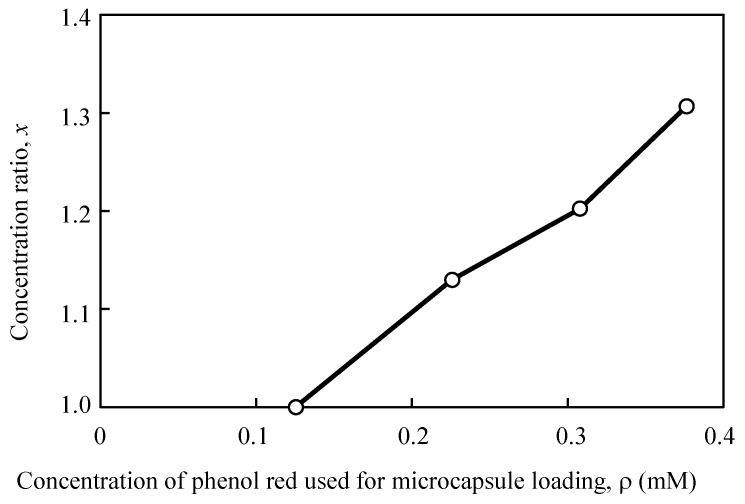
Comparison between the concentration ratio for each phenol red solution loaded into the microcapsules.

Subsequently, the ratio between the encapsulated and the released dye molecule concentration was examined (Figure 6). The released/encapsulated value (*x*) increased in proportion with the encapsulated dye molecule concentration, and the slope calculated from the relationship between them (linear calibration curve) was 1.2 (/mM). This analysis demonstrated that the dye molecules were incorporated in the microcapsules and were subsequently released, and that the concentration of the dye in the release solution changed in proportion with the encapsulated dye concentration. In the application of this amplification method, it is necessary to check the amplitude by measuring a calibration curve according to the density of the target molecules.

## 4. Conclusions

A proof-of-concept of the microcapsule-based amplification method was proposed, which may be adapted for the amplification of biomolecules, such as biomarker analyses, gene analyses, cell analyses, or clinical autoanalyzers. The method outlined in this study demonstrates that the concentration of released molecules from the microcapsules was in proportion with the concentration of encapsulated molecules.

The application of this method may be effective in increasing the sensitivity, enhancing the signal readout, and reducing the size of conventional biosensor systems, such as the biosensor platforms already employed in a microfluidic control mechanism consisting of several chambers connected by flow channels. In particular, paper-based analytical devices, which are very popular for POCT systems where signal amplification strategies are often important to improve sensitivity, are suitable for this microcapsule-based amplification method. Further evaluations of the specific microcapsule-based amplification method would be required for each individual biosensor in order to optimize the performance in line with the specific biomarker and platform under consideration.
